# The Future of Aging in a Longevity Society: A Course for High School and College Students

**DOI:** 10.1093/geroni/igab046.2831

**Published:** 2021-12-17

**Authors:** Paris Strom

**Affiliations:** Auburn University, Auburn University/Auburn, Alabama, United States

## Abstract

Americans, on average, can anticipate living 85 years or perhaps 100 if born in this millennium. This extension of the lifespan has introduced a new stage of human development presenting unfamiliar challenges to policy makers, health care providers, employers, religious institutions, families, individuals, and schools. Education about longevity should begin in adolescence (ages 10-20) with the merger of science, experiences of older generations, and imagination of youth. Content of this online course focuses on the years after adolescence: early adulthood, middle age, retirement, and old age. After reading each of the 16 lessons, cooperative learning teams conduct structured interviews with older relatives, friends or neighbors who are further along in life's journey. All the lessons are augmented by 'what do you think? tasks used to motivate discussions, structure interviews, decide on reasoning and problem-solving scenarios, identify key concepts to apply, group lesson reviews, and self-evaluation for comparison with peers. If society wants to encourage adolescents to appreciate their national and ethnic heritage, benefit from learning how older generations see situations and interpret current events, and acknowledge the common need for maturity and spiritual development, then older people should become resources for education about longevity.

